# Mycotoxins Occurrence in Herbs, Spices, Dietary Supplements, and Their Exposure Assessment

**DOI:** 10.3390/toxins18010020

**Published:** 2025-12-29

**Authors:** Joanna Kanabus, Marcin Bryła, Krystyna Leśnowolska-Wnuczek, Agnieszka Waśkiewicz, Magdalena Twarużek

**Affiliations:** 1 Department of Food Safety and Chemical Analysis, Prof. Wacław Dąbrowski Institute of Agricultural and Food Biotechnology—State Research Institute, Rakowiecka 36, 02-532 Warsaw, Poland; joanna.kanabus@ibprs.pl (J.K.); marcin.bryla@ibprs.pl (M.B.); krystyna.lesnowolska-wnuczek@ibprs.pl (K.L.-W.); 2 Department of Chemistry, Poznań University of Life Sciences, Wojska Polskiego 75, 60-625 Poznań, Poland; 3 Faculty of Biological Sciences, Department of Physiology and Toxicology, Kazimierz Wielki University, Chodkiewicza 30, 85-064 Bydgoszcz, Poland; twarmag@ukw.edu.pl

**Keywords:** mycotoxins, fungi, herbs, spices, dietary supplements, analytical methods, exposure assessment

## Abstract

Mycotoxins are toxic secondary metabolites produced mainly by filamentous fungi of the genera *Aspergillus*, *Penicillium*, and *Fusarium* and pose a significant food safety concern. This review summarizes current literature on the occurrence of major regulated and emerging mycotoxins, including aflatoxins, ochratoxin A, fumonisins, trichothecenes, zearalenone, and selected *Fusarium* and *Alternaria* metabolites, in herbs, spices, and plant-based dietary supplements. Available data indicate that spices—particularly chilli, paprika, ginger, and various types of pepper—represent high-risk commodities and are often more heavily contaminated than dried herbs. Although reported concentrations of individual mycotoxins are frequently low to moderate, numerous studies highlight the common co-occurrence of multiple toxins within a single product, raising concerns regarding cumulative and combined toxic effects. Dietary supplements, especially those containing concentrated plant extracts such as green tea or green coffee, are also identified as potential sources of multi-mycotoxin exposure. The review outlines key analytical approaches for mycotoxin determination, emphasizing the critical role of sample preparation for chromatographic analysis in complex plant matrices. Despite increasing evidence of contamination, important knowledge gaps persist regarding emerging mycotoxins, underrepresented botanical matrices, and long-term exposure assessment, while regulatory limits remain incomplete or inconsistent. Continued monitoring and harmonized analytical and risk assessment strategies are, therefore, essential to ensure consumer safety.

## 1. Introduction

Mycotoxins are a group of approximately 500 toxic secondary metabolites produced by fungi such as *Aspergillus*, *Penicillium*, *Fusarium*, *Claviceps*, and *Alternaria* [1]. These fungi can grow on herbs, spices, and other crops, and their secondary metabolites can pose a threat to human and animal health. The production of mycotoxins often depends on environmental conditions such as humidity, temperature, and the presence of oxygen. The most common mycotoxins found in herbs, spices, and dietary supplements are aflatoxins, ochratoxin A, and fumonisins. These compounds have been confirmed to have potential carcinogenic, teratogenic, and mutagenic effects [1,2,3,4,5]. Their presence contaminates both the crop and the material obtained after harvesting, and the finished product [2,3,4,5]. In addition to the well-recognized problem of single-toxin contamination, recent research has highlighted the frequent co-occurrence of multiple mycotoxins within the same raw material or plant-derived product [6,7]. This phenomenon results from the ability of individual fungal species to synthesize several secondary metabolites, as well as from the simultaneous colonization of plant materials by different filamentous fungi [6]. As a consequence, herbal products, spices, and dietary supplements may contain complex mixtures of chemically diverse mycotoxins, often at low or moderate concentrations. The co-occurrence of multiple contaminants is increasingly regarded as a major challenge for food and herbal safety, as it may influence both the accuracy of analytical detection and the overall toxicological impact on consumers [6,7,8].

Some spices are more exposed to the growth of toxic fungi than herbs. It has been confirmed that spices purchased in bulk packaging from markets are significantly more contaminated than those purchased in supermarkets [8,9]. To minimize the presence of mycotoxins in ready products, it is important to implement appropriate agricultural and manufacturing practices. This includes practicing proper hygiene during the cultivation and processing of plants, as well as ensuring appropriate drying and storage conditions to prevent pathogen growth. Mycotoxins are low molecular weight compounds, weakly polar, and they do not decompose during pasteurization [5,10,11].

The implementation of preventive and control measures against the presence of mycotoxins in plant material is crucial for reducing consumer exposure to these compounds. In recent years, the global burden of mycotoxin contamination has been increasingly recognized in food safety regulation and risk assessment frameworks. For example, the European Food Safety Authority (EFSA) monitors mycotoxin occurrence data and issues opinions on human exposure and health risks [12]. Current legislation in the European Union regulates the presence of selected mycotoxins in selected herbs and spices. Mycotoxin limits have already been established in more than 100 countries [13]. Dietary supplements are a very popular part of everyday life these days. They are supposed to strengthen the body, replenish deficiencies in vitamins and minerals, help fight stress, and improve thought processes [14]. Quality control and regulatory oversight in many jurisdictions remain less rigorous compared to conventional foodstuffs. These factors combined render spices, herbs, and supplements potential vectors of mycotoxin exposure for consumers.

Despite the recognised risk associated with the presence of mycotoxins in cereals and nuts, there is still a lack of comprehensive data on their occurrence in spices, herbs, and dietary supplements. Reviews of spices indicate a high prevalence of mycotoxins and fungi. Regulations often only cover major compounds (such as aflatoxins and ochratoxin A) and exclude many botanical matrices [15]. Similarly, studies on medicinal herbs and plant-based supplements show contamination by multiple mycotoxins and varying degrees of risk, but standardized testing protocols and harmonized regulatory limits are still lacking [16]. In light of these observations, it becomes imperative to examine the full continuum from regulatory quality standards, through fungal contamination, mycotoxin occurrence, analytical methods, to the assessment of consumer exposure risk via these less-studied matrices. The present article provides a comprehensive review of mycotoxin contamination in spices, herbs, and plant-based dietary supplements, with a particular focus on fungal diversity, mycotoxin occurrence and levels, analytical testing methods, and approaches to consumer exposure and risk assessment.

## 2. Presence of Filamentous Fungi and Potential Risk to Human Health

Mycotoxins, products of the secondary metabolism of filamentous fungi, commonly referred to as pathogens, are classified as natural contaminants of food and raw materials used for their production. The presence of these microorganisms and their metabolites in herbs, spices, and dietary supplements is an issue of growing concern for food safety and public health. Approximately 300 to 400 mycotoxins have been identified and reported [17]. Mycotoxins such as aflatoxins, ochratoxin A, fumonisins, and zearalenone have been detected in these products, posing a potential threat to human health. The toxicological relevance of the mycotoxins identified in herbal materials, spices, and dietary supplements is considerable, as these compounds exert a wide range of adverse biological effects [7]. Due to the above, the safety of these products is of interest to scientific centers and consumers. In addition to the toxic effects of one, they are also carcinogenic, mutagenic, teratogenic, and estrogenic. Some common molds that can be found in herbs include *Aspergillus*, *Penicillium*, and *Fusarium* species. The most common toxins found in spices are aflatoxins (produced by *Aspergillus* species) and ochratoxin A (produced by *Aspergillus* and *Penicillium*). The International Agency for Research on Cancer (IARC) classifies aflatoxin B1 as a Group 1 carcinogen [18]. Ochratoxin A exhibits strong nephrotoxic, hepatotoxic, and immunotoxic effects [18]. *Fusarium*-derived metabolites, such as deoxynivalenol and T-2 toxin, act primarily as potent inhibitors of protein synthesis, leading to gastrointestinal toxicity, immunomodulation, and cytotoxicity [16]. Zearalenone is an estrogenic compound that can disrupt endocrine function. Increasing attention has also been directed toward emerging mycotoxins, including enniatins, beauvericin, moniliformin and *Alternaria* toxins, which display cytotoxic, ionophoric, genotoxic, and in some cases, estrogenic activities [16,17]. Notably, many herbal products contain multiple mycotoxins simultaneously, which raises additional toxicological concerns due to the possibility of additive or synergistic effects. Such combined exposure may enhance overall toxicity, even when the concentrations of individual toxins remain low, underscoring the need for integrated risk assessment that accounts for cumulative mycotoxin burden in plant-derived products. Selected isolated fungal species and the mycotoxins they produce are presented in Table 1.

Proper handling, storage, and quality control measures are essential to prevent mold growth and mycotoxin contamination in herbs [17,18,19]. Mycotoxin contamination of food and feed depends significantly on environmental conditions that may enable and accelerate the formation and growth of mold. Contamination may occur at any stage of production (plant development, collection, processing, storage, and transport) [30,31,32]. Physical methods applied in technological food processing use high or low temperatures, microwave radiation, ultrasonic waves, high pressure, or infrared radiation, and are considered among the most effective means of reducing toxin levels in contaminated materials [33,34].

Diseases called mycoses are among the direct disease effects caused by fungi. They cause generalized and organ (invasive) mycoses, as well as sensitization. Fungal allergens (spores and fungal fragments) are carried with air bioaerosols. After entering the respiratory system and remaining there, especially in individuals and animals prone to sensitization, they can cause reactions such as bronchial thrush, conjunctivitis, allergic rhinitis, and even alveolitis [35].

Both our own studies and those conducted by global scientific centers confirm that dangerously high levels of contamination by mold are present in herbs, medicinal plants, as well as dietary supplements, which can pose a threat to human health. A 2009 study by Tournas found mold and yeast in herbal products and dietary supplements. The most common molds belonged to the genera *Aspergillus*, *Penicillium*, and *Eurotium* multiple species within the genus. The highest amount of mold and yeast was found in supplements containing *Medicago sativa* L. (5.6 × 106 cfu/g), while the lowest amount was found in supplements containing ginger (1.0 × 102 cfu/g) [36]. Another study conducted by Filipiak-Szok [37] looked at the contamination of dietary supplements and plant extracts, such as ginseng, Chinese angelica, and *Withania somnifera* (ashwaganda). The results confirmed high levels of contamination by both mycotoxins and mold (*Cladosporium*, *Eurotium*, *Aspergillus*, *Rhizopus*, and *Penicillium* multiple species within the genus). Green coffee and its extracts are counted as dietary supplements that aid weight loss. Previously, they were not counted as products that endanger human health and life, as they were not consumed in this form. A study conducted by Twarużek et al. (2015) [38] showed significant contamination of green coffee samples. The tested material was mainly dominated by *Aspergillus* molds (63%) and *Penicillium* (25%), whileF coffee extract was contaminated mainly with *Penicillium* multiple species within the genus (72%). Rajeshwari et al. (2016) [39] tested herbal preparations for the presence of, among other things, molds. They tested 18 herbal substances, including calamus, senescence, gotu kola, muscatel, guduchi, and ashwagandha. Only 6 of the tested materials recorded the presence of fungi. The most frequently isolated molds belonged to the genera *Aspergillus* (20%) and *Penicillium*, multiple species within the genus (17%).

Presence of fungal pathogens is a widespread and multifactorial problem in the production chain of herbs, spices, and dietary supplements. It may occur both in the field and during post-harvest stages, and the diversity of fungal species involved increases the likelihood of multiple mycotoxins co-occurring in a single product. Implementing comprehensive quality control, proper drying and storage procedures, and continuous microbiological monitoring is therefore essential to ensure the safety of these widely used plant-based commodities.

## 3. Occurrence of Mycotoxins

Mycotoxins are widely distributed in different products such as cereal, nuts, fruits and dry fruits, vegetables, kelp, wines, meat, juice, coffee, and milk [39,40,41]. Occurrence of mycotoxins depends on several environmental and technological factors, such as climatic and geographical conditions, agricultural practices, humidity, soil pH, and storage methods [32]. Contamination of raw materials can occur during cultivation, harvesting, and storage. Many mycotoxins are highly thermally stable and resistant to processing, so even after visible signs of fungal growth have been removed, they may remain in the final product [32]. Herbal raw materials, spices, and dietary supplements often contain different species of fungi, which promotes the co-occurrence of multiple mycotoxins in a single product. This phenomenon is particularly significant because a single species can synthesise several toxins, and different species can colonise the same raw material [39,40]. As a result, plant products may contain complex mixtures of classic toxins (aflatoxins, ochratoxin A, zearalenone, deoxynivalenol, fumonisins), and emergent toxins such as enniatins, beauvericin, moniliformin, and alternariol. Since the presence of mycotoxins does not always correlate with visible fungal growth, regular chemical analyses are necessary to assess the compliance of products with applicable safety standards [42,43].

To reduce exposure to mycotoxins potentially present in food, European Union regulations establish maximum permissible levels for aflatoxins, ochratoxin A, patulin, zearalenone, deoxynivalenol, and fumonisins in cereal grains, other plant raw materials, and processed products. These limits are designed to ensure that the level of contamination in food remains as low as reasonably achievable. Identification of fungal species present in raw materials plays a crucial role in assessing contamination risk. Accurate identification of individual species of mold determines the degree of contamination and allows prediction of potential mycotoxin production [39,40,42,43].

Some research has suggested that certain herbal raw materials may not provide favorable conditions for mycotoxin biosynthesis, possibly due to the presence of essential oils with antifungal activity. Velazhahan et al. [44] observed that herbal raw materials are not an environment conducive to the production of mycotoxins, suggesting that the presence of essential oils may inhibit the biosynthesis of these compounds. The inhibitory effect of some oils or plant extracts, e.g., *Trachyspermum ammi*, *Cinnamomum camphora*, *Thymus vulgaris*, *Citrus aurantifolia*, *Mentha spicata*, on the growth of molds and the production of aflatoxins was observed to varying degrees [44].

Herbal plants represent a diverse group of materials obtained for medicinal, culinary, cosmetic, and feed purposes. Research related to the microbiological assessment of herbal materials and spices shows that among biological factors, a significant proportion of these products are contaminated with fungal microflora filaments [45].

It is important to note that the presence of mycotoxins in herbs can vary depending on factors such as storage conditions, moisture content, and exposure to fungi during growth and processing. Proper handling, storage, and regular analytical testing of herbs can help minimize the risk of mycotoxin contamination [44,45,46,47,48].

As a class, mycotoxins are low-molecular substances (300–600 Da) for which the body does not produce antibodies. They are extremely resistant to environmental factors and can accumulate in tissues and organs, leading to mycotoxicosis. Chronic exposure may result in severe diseases or death [49]. Depending on their toxicological effects, mycotoxins are classified as pneumotoxins (causing pulmonary edema), dermatotoxins (leading to damage to the skin and mucous membranes), nephrotoxins, cardiotoxins, hepatotoxins, and neurotoxins. Numerous studies have confirmed the occurrence of various mycotoxins in herbs, spices, and dietary supplements. Examples of herbs, spices, and plant-based dietary supplements in which the presence of selected mycotoxins has been observed are presented in Table 2.

The data summarized in Table 2 indicate that herbs, spices, and plant-based dietary supplements are frequently contaminated with a broad spectrum of mycotoxins, including both compounds regulated by current legislation and emerging toxins. Among the commonly detected contaminants were aflatoxins and ochratoxin A, which appeared in numerous herb samples (such as mint, thyme, valerian and coriander) as well as in spices and multi-component herbal mixtures. Their concentrations ranged from the limit of detection to values exceeding established safety limits. In addition, *Fusarium* metabolites—including zearalenone, trichothecenes, fumonisins and a wide array of emerging toxins such as enniatins and beauvericin—were identified in various herbal products, sometimes at notably high concentrations, particularly in milk thistle (*Cardu marianus*), boldus and certain spice matrices. Spices represented the most heavily contaminated group of products, with chilli, paprika, ginger and different types of pepper exhibiting elevated levels of aflatoxins, ochratoxin A, fumonisins and, in some cases, sterigmatocystin. Dietary supplements, especially those containing green tea or green coffee bean extracts, frequently showed multi-toxin contamination, with concurrent detection of ochratoxin A, fumonisins, sterigmatocystin, enniatins and aflatoxins. The observed patterns clearly demonstrate that plant-derived materials may contain complex mixtures of mycotoxins originating from multiple fungal genera, highlighting the importance of routine monitoring and consideration of combined exposure in the risk assessment of herbal products and supplements. The co-occurrence of multiple mycotoxins—even at low levels—poses a potential cumulative toxicological risk. Continued monitoring, harmonized analytical methodologies, and stricter regulatory oversight are therefore essential to ensure consumer protection and product quality.

## 4. Requirements and Standard of Quality

Quality standards and safety requirements for herbs, spices, and dietary supplements vary considerably worldwide, depending on whether products are regulated as food, traditional herbal medicines, or dietary supplements [64]. In the European Union (EU), herbal products and spices are subject to Regulation (EC) No 178/2002 establishing general principles of food law, and Regulation (EC) No 915/2023, which sets maximum levels for certain contaminants, including mycotoxins, in foodstuffs [65,66]. These limits primarily apply to cereals, nuts, dried fruits, and baby food, while only a few cases are regulated for dried spices. According to the European Union (EU) standard, the content of aflatoxin B_1_ in spices such as pepper, paprika, ginger, and a mixture of spices is 5 µg/kg, and the sum of aflatoxins (AFB1, AFB2, AFG1, AFG2) in spices is 10 µg/kg (European Commission, 2023) [64,65,66]. For ochratoxin, the maximum OTA content for paprika and pepper is 20 µg/kg, and for other dried spices and ginger it is 15 µg/kg [66].

In the United States, dietary supplements are regulated under the Dietary Supplement Health and Education Act, which defines them as a category of food [66]. Contaminant limits, including for mycotoxins, follow guidance from the U.S. Food and Drug Administration (FDA), such as limits for aflatoxins in food and feed [67]. In Asia, particularly in China, India, and Japan, national pharmacopeias define quality parameters for herbal raw materials, yet limits for mycotoxins are inconsistently applied or absent for many herbal ingredients [68]. The European Pharmacopoeia requires that herbal drugs intended for oral use must be tested for total aflatoxins (≤4 µg/kg) when contamination risk is suspected [67,68]. However, these tests are not mandatory for all plants, and coverage of other major mycotoxins (e.g., ochratoxin A, fumonisins, zearalenone) is limited. Dietary supplements, on the other hand, often contain mixtures of herbal extracts, powders, and plant-based concentrates, making it difficult to apply a uniform standard. Good Manufacturing Practice (GMP) systems (e.g., ISO 22000, WHO Guidelines for Herbal Medicines, or FDA GMP for dietary supplements) require monitoring of microbial and chemical contaminants, but analytical verification for mycotoxins is often voluntary or limited to high-risk materials [66,67,68,69].

One of the main challenges lies in the lack of harmonized regulatory limits for mycotoxins in all products. While the European Union and the United States have well-defined limits for major food categories such as cereals and nuts, comparable values are absent or inconsistently applied for herbs and dietary supplements. As a consequence, the same local product may meet safety requirements in one country but exceed acceptable levels in another. A further limitation is that routine quality control procedures in the herbal industry are primarily focused on the parameters such as identity, purity and microbiological quality, while chemical contaminants, including mycotoxins, are often overlooked. Analytical screening for aflatoxins or ochratoxin A is typically performed only when contamination is suspected, rather than as part of systematic quality assurance. Another issue concerns analytical variability. Differences in sample preparation, extraction efficiency, and method sensitivity among laboratories lead to inconsistent results, making cross-study comparisons difficult. Limited data from official monitoring programs contribute to the uncertainty surrounding consumer exposure. In many regions, particularly outside the EU, there are no mandatory reporting systems for mycotoxin levels in herbal products. Finally, the classification of certain products is difficult. Herbal teas, botanical blends, and plant-based supplements may be regulated differently depending on their labeling or intended use, resulting in unequal safety standards and loopholes in quality control. International collaboration between food safety authorities, pharmacopoeial bodies, and public health agencies is essential to ensure that these widely consumed products meet equivalent safety and quality standards worldwide [64,65,66,67,68,69].

## 5. Mycotoxins Testing Methods

At present, mycotoxins research is mainly focused on its toxicity occurrence and the improvement of detection methods. Most mycotoxins are toxic at very low concentrations; therefore, it is necessary to use sensitive and reliable analytical methods is essential for their detection. The determination of mycotoxins, generally relies on their physicochemical properties and involves extraction with appropriate solvents (e.g., methanol, acetonitrile), followed by purification of extracts and qualitative and quantitative analysis. Although numerous studies have been devoted to mycotoxin detection in food and feed, research concerning natural plant-based matrices such as herbs and supplements remains limited but continues to develop dynamically. This section summarizes both traditional methods and modern instrumental approaches such as mass spectrometry [70,71,72,73,74].

Extraction is a critical step in mycotoxin analysis, as it enables the isolation of analytes from complex matrices containing lipids, proteins, and other interfering substances. Popular analytical methods used for mycotoxin isolation include solid–liquid extraction (SLE), liquid-liquid extraction (LLE), matrix solid phase extraction (MSPD), solid-phase extraction (SPE), and solid phase microextraction (SPME) [75]. The QuEChERS method (Quick, Easy, Cheap, Effective, Rugged and Safe) is widely applied for mycotoxin determination in various plant materials, including fruits, vegetables, cereals, spices and herbs. It combines solvent extraction with dispersive solid-phase purification, ensuring efficient and repeatable results [71,76,77,78,79].

To remove matrix interferences, different sorbents are used during purification, such as modified silica gels, PSA (primary secondary amine), activated carbon, GCB (graphitized carbon black), florisil, diatomaceous earth, or chitin [80].

Moreover, modern extraction methods such as microwave-assisted extraction (MAE) and ultrasound-assisted extraction (UAE) have gained popularity due to their efficiency and reduced solvent use. Other advanced methods include accelerated solvent extraction (ASE), supercritical fluid extraction (SFE), and vortex-assisted low-density solvent microextraction (VALDS-ME) [81,82,83,84].

Among purification approaches, immunoaffinity columns (IACs) and molecularly imprinted polymers (MIPs) play an important role. IACs provide highly selective purification and generate clean extracts with minimal matrix interference, although they require aqueous samples and specialized handling [32,85]. MIPs, in turn, are synthetic materials that mimic biological recognition sites, allowing specific binding of mycotoxins. These polymers are created through copolymerization in the presence of a template molecule (e.g., mycotoxin), which is later removed, leaving binding cavities complementary in shape and functionality to the analyte [85,86,87]. Despite progress in extraction techniques, it is worth emphasizing that sample preparation remains the largest source of analytical error, due to the complex biochemical composition of plant matrices and matrix effects that can influence detection results [81,88,89]. Chromatographic and non-chromatographic methods used for mycotoxin analysis in herbs, spices, and dietary supplements are shown in Table 3.

Thin-layer chromatography (TLC) was historically the most common method used in mycotoxin analysis. TLC has largely been replaced by high-performance liquid chromatography (HPLC) and, more recently, ultra-performance liquid chromatography (UPLC), which provides better resolution, higher sensitivity, and shorter analysis times [89]. HPLC coupled with fluorescence detection (HPLC-FLD) remains a standard method for mycotoxin testing, but LC-MS/MS systems have revolutionized the field by enabling multi-residue detection of numerous mycotoxins in a single run—up to 35 different compounds in plant matrices [90,91]. Gas chromatography (GC) is also employed, particularly for volatile or derivatized compounds, but liquid chromatography (LC) offers an advantage in analyzing thermally unstable or non-volatile substances [51,92]. The coupling of chromatographic methods with mass spectrometry (MS)—including GC-MS/MS and LC-MS/MS—has become the gold standard for mycotoxin detection. Mass spectrometers serve as highly sensitive, selective, and universal detectors, identifying compounds by their mass-to-charge (*m*/*z*) ratios and providing structural information through fragmentation patterns. In mycotoxin analysis, quadrupole (single or triple), ion trap (IT), and time-of-flight (TOF) analyzers are commonly used [71,77].

In addition to chromatographic techniques, immunochemical and molecular methods offer rapid and cost-effective alternatives for screening purposes. Enzyme-linked immunosorbent assays (ELISA) are among the most popular tools for detecting regulated mycotoxins, providing good sensitivity and quantitative capability. Commercial ELISA kits are widely available and validated for aflatoxins, ochratoxin A, fumonisins, zearalenone, and trichothecenes, operating on a competitive format where signal intensity is inversely proportional to antigen concentration [32,93,94,95,96,97]. Lateral Flow Immunoassays (LFIAs), also known as strip tests, are another rapid diagnostic method based on immunochromatography, allowing qualitative or semi-quantitative detection of mycotoxins in minutes [97,98]. These rapid tests are particularly valuable for on-site screening, enabling preliminary identification of contaminated batches without sophisticated equipment. Emerging molecular approaches include DNA-based assays and aptamer-affinity systems, which can identify toxigenic fungal strains rather than the toxins themselves, providing early warning of potential contamination.

Based on the literature, analytical techniques for mycotoxin determination are typically evaluated according to three main criteria: analytical performance, speed of analysis and cost [99,100]. Overall, continuous improvement in analytical methods is necessary to enhance food and herbal product safety, reduce consumer exposure, and meet regulatory expectations.

## 6. Risk Assessment of Mycotoxin Intake

Recent studies conducted around the world highlight the fact that people are increasingly exposed to the natural co-occurrence of mycotoxins in food and the globalization of the food market [101]. Climatic changes, such as increases in temperature or humidity in some regions of Europe, can promote the growth of fungal pathogens, thereby increasing the likelihood of mycotoxin contamination of food products [102]. As a result, this poses a serious threat to human and animal health. To determine the degree of risk of mycotoxins entering the body from food, an exposure assessment is performed, which is one of the steps in risk estimation. Its purpose is to determine the dose or concentration of a chemical compound that is harmful to humans [103].

In the past, health risks from human exposure to chemical contaminants were assessed based on single-substance and single-exposure pathway scenarios. In recent years, however, the World Health Organization (WHO) and the European Food Safety Authority (EFSA) have proposed a tiered approach to assess the risks of multiple chemicals, including contaminants. This hierarchical approach includes integrated and incremental exposure and hazard considerations at all stages, with each tier being more refined (less conservative and uncertain) than the previous one, requiring expanded knowledge of the group of chemicals being evaluated and their mixtures. This has led to the concept of combined toxicity, which is defined as the response of the biological system to several chemicals following simultaneous or sequential exposures [104]. Combined toxicity can take three possible forms: concentration addition (CA), independent action (IA), and interaction [105]. According to the CA model, the combined action of multiple chemicals is the sum of individual toxicities, assuming the same mode of action (MoA), in the same cell, tissue, or target organ. In contrast, the IA model assumes that the combined effects are estimated with independent effects of chemical compounds, by different MoA or in different cells, tissues or organs [106,107].

These two reference models have been successfully applied in toxicological evaluations, mixtures of similarly acting and differently acting compounds, both in ecotoxicological studies using a range of species [108,109] and in human toxicity studies using cell lines or animal models [110]. Deviations from these patterns include synergism (mixture effect greater than additive), antagonism (mixture effect less than additive), and more subtle interactions that depend on the actual doses of the mixture components [107].

Human risk assessment is based on a four-step process, namely:

- hazard identification—identifying the chemical compound that poses a health risk;
- hazard characterization—the conditions under which a specific chemical compound may cause adverse health effects or disease, and the dose;
- exposure assessment—estimating the frequency, intensity and duration of ingestion of the toxin;
- risk characterization—integrating the results of the exposure assessment with the results of the risk characterization to estimate the degree of concern [103].

Hazard identification and risk characterization allow us to make an exposure assessment, which is an important point in risk assessment. In this step, we use information on the amount of food consumed per unit of time (day, week) and body weight. We can perform the calculation in two ways: deterministic, in which we consider the values of the variables, i.e., the mean or median; probabilistic, otherwise known as Monte Carlo simulation, in which we use randomly selected values of variables, giving us a probability distribution. The final step in risk assessment is risk characterization, which is based on a quantitative or qualitative assessment of the probability of adverse health effects associated with the ingestion of a chemical compound [111,112]. For risk characterization, hazard quotients (HQs) are commonly derived by comparing estimated exposure levels with established toxicological reference values, including the no-observed-adverse-effect level (NOAEL), the no-observed-adverse-effect concentration (NOAEC), and the lowest-observed-adverse-effect level (LOAEL). NOAEL represents the highest dose at which no adverse effects are observed, while NOAEC refers to the highest tested concentration without observable adverse effects, typically used for inhalation or environmental exposure assessments. LOAEL is defined as the lowest dose or concentration at which adverse effects are detected. An HQ value below 1 indicates acceptable exposure, whereas an HQ greater than 1 suggests a potential health concern [111,112].

## 7. Summary

Herbal raw materials, spices, and plant-based dietary supplements represent a significant source of exposure to both regulated and emerging mycotoxins. The studies analyzed show that these products are frequently contaminated with aflatoxins, ochratoxin A, zearalenone, fumonisins, deoxynivalenol, nivalenol, as well as emerging toxins such as enniatins, beauvericin and *Alternaria* metabolites. Among the reviewed commodities, spices—particularly chilli, ginger, and various types of pepper—exhibited the highest contamination levels. In contrast, herbal products and plant-based dietary supplements often contain multiple mycotoxins simultaneously, increasing the potential for additive or synergistic toxic effects. Due to the high thermal stability and persistence of these contaminants, reliable analytical control is essential. Modern chromatographic techniques, especially LC–MS/MS combined with QuEChERS extraction and selective purification, enable sensitive detection of multiple mycotoxins in complex plant matrices. Rapid screening tools such as ELISA and lateral flow assays complement instrumental analysis. Overall, the results clearly indicate the need for continuous monitoring and improved analytical strategies to ensure consumer safety and effective management of toxicological risks associated with plant products.

## Figures and Tables

**Table 1:** Selected species of filamentous fungi, the types they represent, examples of the mycotoxins they produce, and their potential risk to human health.

Species of Filamentous Fungi	Name of Isolated Strain	Examples of Produced Mycotoxins	Potential Risk to Human Health	References
*Fusarium* sp.	*F. proliferatum*	fumonisins B1, B2, B3	Carcinogenic, hepatotoxic, nephrotoxic	[16]
		beauvericin	Cytotoxic	[19,20]
		enniatins (B, B1, A, A1)	Cytotoxic with the capacity to disrupt intracellular ionic homeostasis	[16,20]
		fusaproliferin	Cytotoxic	
		moniliformin	Carcinogenic, weight loss, intestinal haemorrhage	
	*F. avenaceum*			
	*F. temperatum*			
	*F. tricinctum*			
	*F. graminearum* *F. culmorum*	deoxynivalenol	Inhibits DNA, protein synthesis, and causes immunosuppression.	[16,21,22]
		3-acetyl-deoxynivalenol	Apoptosis, inhibition of cell division, disruption of DNA repair processes.	[22]
		15-acetyl-deoxynivalenol		
		nivalenol	Inhibition of cell division	[23]
		fusarenon-X	Inhibition of protein synthesis	[24]
		zearalenone	Estrogenic activity	[16]
		α-zearalenol β-zearalenol	Increases oestrogenic activity	[25]
	*F. sporotrichioides*	T-2 toxin	Inhibitor of protein synthesis and mitochondrial function in vitro and in vivo; immunosuppressive and cytotoxic effects	[16,26]
		HT-2 toxin	Dermatotoxic, ecrosis and bleeding of the intestinal mucosa	
		4,15-diacetoxyscirpenol	Hepatotoxic	[27]
	*F. poae*			
		beauvericin	Cytotoxic	[16,19,20]
		enniatins (A, A1, B, B2)	Cytotoxic with the capacity to disrupt intracellular ionic homeostasis	
	*F. tricinctum*			
*Aspergillus* sp.	*A. flavus* *A. parasiticus* *A. nomiae*	aflatoxins B1, B2, G1, G2	B1 classified as group 1 of human carcinogens	[16,28]
	*A. ochraceus* *A. carbonarius* *A. niger* *A. westerdijkiae* *A. steynii*	ochratoxin A	Strong nephrotoxic, hepatotoxic and immunotoxic effects	[16,18]
	*A. flavus*	cyclopiazonic acid	Carcinogenic, weight loss, loss of lactation	[16]
	*A. versicolor*	sterigmatocystin	Carcinogenic, diarrhoea, changes in the liver and kidneys	[16]
*Penicillium* sp.	*P. verrucosum* *P. nordicum*	ochratoxin A	Strong nephrotoxic, hepatotoxic and immunotoxic effects	[16,18]
	*P. patulinum* *P. urticae* *P. chrysogenum* *P. expansum*	patulin	Pulmonary and cerebral haemorrhages	[16]
	*P. citrinum* *P. verrucosum*	cytrinin	Nephrotoxic	[29]
	*P. citreoviride* *P. ochrosalmoneum* *P. toxicarium*	cytreoviridin	Nephrotoxic, impairs the functioning of mitochondria	[16]
	*P. islandicum*	luteoskyrin	Mutagenic	[16]
	*P. cyclopium*	penicillic acid	Less toxic than patulin	[16]
*Alternaria* sp.	*A. alternata*	alternaliol alternariol monomethyl ether tenuazonic acid altenuene	Inhibits cell division and induces cell apoptosis, mimics the action of hormones	[30]
	*A. arboerscens* *A. tenuissima*			

**Table 2:** Examples of mycotoxin content in analysed samples of spices, herbs, and plant-based dietary supplements.

Pant Material	Identified Mycotoxins	Concentration [µg/kg]	References
Herbs			
Pennyroyal mint (*Mentha pulegium* L.) (water infusion)	aflatoxin B2 aflatoxin G2	71.9 (mean) 3.8 (mean)	[50]
Mint (*Mentha* sp.)	ochratoxin A aflatoxin zearalenone t-2 toxin deoxynivalenol citrinine	1–1.4 16.6–29.7 2.1–9.3 3.9–4.9 46.9–91.1 41.0–43.3	[51]
	aflatoxins ochratoxin A	<LOD-0.89 <LOD	[52]
Thyme (*Thymus vulgaris* L.)	aflatoxin B1 ochratoxin A	0.72–0.96 0.02–1.94	[52]
Thyme (*Thymus vulgaris* L.) (water infusion)	aflatoxin B2 aflatoxin G2	112.2 (mean) 4.7 (mean)	[50]
Valerian (*Valeriana officinalis* L.) (water infusion)	aflatoxin G2	13.5 (mean)	
Valerian (*Valeriana officinalis* L.)	enniatin A enniatin A1 enniatin B enniatin B1	63.1–88.7 8.5–42.7 0.8–27.8 22.3	[16]
Horsetail (*Equisetum arvense* L.) (water infusion)	aflatoxin G2	2.2 (mean)	[50]
Tea/Green Tea (*Camellia sinensis* L.) (water infusion)	aflatoxin B2	14.4–32.2	
Herbal mix for insomnia (species not specified)	ochratoxin A enniatin A	799 (mean) 3.8 (mean)	[16]
Milk thistle (*Silybum marianum* (L.) *Gaertn.*)	enniatin A enniatin A1 enniatin B enniatin B1 beauvericin	36.6–109.2 57.6–534.9 6.2–1378.2 24.2–1165.9 <LOQ-542.7	
Boldus (*Peumus boldus Molina*)	zearalenone enniatin B	1169–1995 1.8–5.4	
Melissa (*Melissa officinalis* L.)	zearalenone enniatin B	117 (mean) 6.6 (mean)	
Chamomile (*Matricaria chamomilla* L.)	fumonisins	20–70	[53]
	ochratoxin A	0.8–1.0	[51]
	fumonisins	<LOD-90	
	aflatoxins	35.8–161	
	zearalenone	7.3–12.5	
	t-2 toxin	3.5–8.3	
	deoxynivalenol	123.4–191.5	
	cytrinina	31.7–38.9	
	aflatoxins	3.4–38.9	[54]
Coriander (*Coriandrum sativum* L.)	aflatoxin B1 ochratoxin a	0.85–2.16 0.02–10.98	[52]
Oregano (*Origanum vulgare* L.)	aflatoxin B1 ochratoxin A	<LOD-0.82 0.67–4.59	
	fumonisin B1 fumonisin B2 ochratoxin A	6.83 (mean) 5.33 (mean) 22.12 (mean)	[55]
Basil (*Ocimym basilicum* L.)	aflatoxin B1 ochratoxin A	<LOD 0.45–0.71	[52]
Anise (*Pimpinela anisum* L.)	aflatoxin B1 ochratoxin A	0.84–4.87 0.02–23.82	
Rosemary (*Rosmarinus officinalis* L.)	fumonisin B1 fumonisin B2 ochratoxin A	7.72 (mean) 8.29 (mean) 5.07 (mean)	[55]
Thyme (*Thymus vulgaris* L.)	ochratoxin A	15.59 (mean)	
Spices			
Ginger (*Zingiber officinale Roscoe*)	zearalenone enniatin B beauvericin	3850 (mean) 3.3–15.1 95.7–136.8	[16]
Herbal medicines	total aflatoxins aflatoxin B1	4.5–108.4 11.9–73.3	[33]
Curry (species not specified)	fumonisin B1 ochratoxin A	6.56 (mean) 19.01 (mean)	[55]
Paprika/Red pepper (*Capsicum annuum* L.)	ochratoxin A aflatoxin B1	3.0–12.0 <LOD	[56]
	fumonisin B1 sterigmatocystin	11–124 <LOD	[57]
	aflatoxin B1 ochratoxin A	0.15–0.94 0.13–3.62	[52]
Sweet pepper (*Capsicum annuum* L.)	aflatoxin B1 ochratoxin A	0.14–0.96 0.47–44.22	[52]
White pepper (*Piper nigrum* L.)	aflatoxin B1 ochratoxin A	0.15–3.11 0.12–452.46	[52]
	fumonisin B1 fumonisin B2 ochratoxin A	7.20 (mean) 5.28 (mean) 29.41 (mean)	[55]
Black pepper (*Piper nigrum* L.)	aflatoxin B1 ochratoxin A	0.15–0.32 0.85–238.95	[52]
	fumonisin B1 fumonisin B2 ochratoxin A	9.77 (mean) 9.03 (mean) 9.46 (mean)	[55]
Red chilli pepper (*Capsicum annuum* L.)	total aflatoxins aflatoxin B1	0.13–57.30 0.07–55.90	[58]
	total aflatoxins aflatoxin B1	0.19–19.89 0.38–22.24	[28]
Nutmeg (*Myristica fragrans Houtt.*)	aflatoxin B1 ochratoxin A	1.65–18.35 1.39–236.26	[52]
Cinnamon (*Cinnamomum* sp.)	aflatoxin B1 ochratoxin A	0.15–0.84 0.02–2.99	[52]
	ochratoxin A	2.14 (mean)	[55]
Cumin (*Cuminum cyminum* L.)	aflatoxin B1 ochratoxin A	0.14–1.90 0.12–31.99	[52]
Dietary supplements			
Green tea (*Camellia sinensis* (L.) *Kuntze*)	aflatoxin B1	5.4 (mean)	[59]
Plant-based (various plant species)	ochratoxin A	<1.16–20.23	[60]
	fumonisin B1	34 to 524	[34]
	T-2 toxin	64 (mean)	[61]
Dietary supplements containing green coffee bean extracts (*Coffea arabica* L.)	ochratoxin A ochratoxin B fumonisin B1 mycophenolic acid	<1.0–136.9 <1.0–20.2 <50.0–415.0 <5.0–395.0	[62]
Green tea (*Camellia sinensis* (L.) *Kuntze*)	ochratoxin A, fumonisin B2, sterigmatocystin, beauvericin enniatin A aflatoxin B1	12.2 (mean) 76.3 (mean) 19.8 (mean) 4.4 (mean) 1.7 (mean) 1.2 (mean)	[63]

**Table 3:** Chromatographic and non-chromatographic methods used for mycotoxin analysis in herbs, spices, and dietary supplements.

Method Group	Technique	Application	Advantages	Limitations	Example of Matrices	References
Purification and sample preparation methods						
Basic extraction methods	SLE—Solid–Liquid Extraction	Broad-spectrum extraction of mycotoxins	Simple, low-cost	High solvent consumption, low selectivity	Dried herbs, spices, herb tablets	[75]
	LLE—Liquid–Liquid Extraction	Pre-cleaning of extracts	Good separation from lipids	Time-consuming, analyte loss possible	Supplements in the form of plant oils	
Advanced extraction	MSPD—Matrix Solid Phase Dispersion	Extraction from difficult matrices	Combines extraction & cleanup	Requires sorbent optimization	Hard spices and herbs	
	SPE—Solid Phase Extraction	Cleanup before LC-MS/HPLC	High selectivity	Requires sorbent choice	Powdered herbs, plant supplements, finely ground spices	
	SPME—Solid Phase Microextraction	Volatile/semi-volatile toxins	Solvent-free	Low efficiency for large analytes	cinnamon, aniseed, cloves; essential oils	
Modern extraction	QuEChERS	Multi-mycotoxin extraction in complex matrices such as herbs, spices, cereals, and supplements	Fast, low-cost, minimal solvent use, suitable for LC-MS/MS, compatible with many matrices	Requires optimization of sorbents (PSA, C18), potential loss of planar molecules, matrix effects may remain	Spice mixes, chilli, curry, ginger, dried herbs, supplements in capsules and tablets	[71,76,77,78,79]
	MAE—Microwave Assisted Extraction	Accelerated extraction	Fast, reduced solvents	Thermal degradation risk	Dried herbs, ginger, turmeric, hard spices	[81,82,83,84]
	UAE—Ultrasonic Assisted Extraction	Extraction from raw matrices	Fast, inexpensive	Inconsistent efficiency possible	Fresh and dried herbs, powdered supplements, spice mixes	
	ASE—Accelerated Solvent Extraction	Pressurized solvent extraction	High efficiency	Expensive instrumentation	Supplements from plant extracts, green coffee, bark and roots	
	SFE—Supercritical Fluid Extraction	Plant material extraction	Green method, low solvent use	Limited availability	Oil-based supplements, spices rich in essential oils	
Selective cleanup	IAC—Immunoaffinity Columns	Cleanup of aflatoxins, ochratoxin A, zearalenone	Highly selective	Costly, requires aqueous extracts	All spices, herbs and herbal supplements	[32,85,87]
	MIPs—Molecularly Imprinted Polymers	Selective toxin binding	High specificity	Optimization required	Herbal supplements, ground spices, herbal teas	
Instrumental methods						
Chromatography	HPLC-FLD (High-performance liquid chromatography with fluorescence detection)	aflatoxins, ochratoxin A	High sensitivity	Limited multi-toxin capability	All spices and herbs, plant supplements, herbal mixtures	[71,77,89,90,91,92]
	LC-MS/MS (Liquid chromatography-tandem mass spectrometry)	multi-mycotoxin analysis	Gold standard; high selectivity	Expensive instruments		
	GC-MS/MS (Gas chromatography-tandem mass spectrometry)	patulin, volatile trichothecenes	High selectivity	Requires derivatization	Aromatic spices with volatile fractions	
Immunochemical screening	ELISA (Enzyme-linked immunosorbent assay)	aflatoxins, ochratoxin A, fumonisins, deoxynivalenol	Fast, inexpensive	Cross-reactivity	Ground spices, single herbs, powdered supplements	[32,92,93,94,95,96,97,98]

## Data Availability

No new data were created or analyzed in this study.
